# Pathogenesis, diagnosis and treatment of primary renal well-differentiated neuroendocrine tumors: a review of the literature

**DOI:** 10.3389/fonc.2024.1298559

**Published:** 2024-10-04

**Authors:** Zhongqi Zhang, Chenming Luo, Tengfei Yuan, Pinxu Ge, Faping Li, Yanpeng Fan, Yuchuan Hou

**Affiliations:** Department of Urology, First Hospital of Jilin University, Changchun, China

**Keywords:** neuroendocrine, kidney, uro-oncology, oncology, pathogenesis

## Abstract

Neuroendocrine tumors (NETs) are a rare type of neoplasm that originate from neuroendocrine cells and peptide neurons. Primary renal well-differentiated NETs are extremely rare, and only a few cases have been reported worldwide. In this study, we present a new case of primary renal well-differentiated NET at our institution, followed by a literature review. A systematic search was conducted using various search terms to identify relevant literature on primary renal well-differentiated NETs from 2021 to present. The study analyzed the clinical features, age, gender, tumor size, location, gross pathology, light microscopy, and immunohistochemical results of 32 cases of primary renal well-differentiated NETs. The findings suggest that these tumors are rare and have nonspecific clinical and imaging features. The diagnosis heavily relies on immunohistochemical analysis. Primary renal well-differentiated NETs are associated with low malignant potential and a favorable prognosis. Surgical resection is the preferred treatment, and long-term follow-up is necessary to monitor the patient’s condition.

## 1 Introduction

Neuroendocrine tumors (NETs) arise from neuroendocrine cells and peptide neurons, and are typically found in the respiratory and gastrointestinal tracts. While NETs are a rare type of tumor in the genitourinary system, over 160 cases have been reported worldwide as of 2023. In this study, we present a case of primary renal well-differentiated NET and provide a literature review of related cases.

## 2 Materials and methods

A 55-year-old female patient was found to have a left renal tumor during a routine physical examination. Multi-phase enhanced CT revealed a heterogeneous soft tissue density mass in the left kidney, with significant heterogeneous enhancement during the arterial phase, decreased density during the venous and pyelographic phases, and non-enhanced areas within, measuring approximately 6.9 × 7.1 cm in size. A low-density round lesion with Bosniak classification of IIF was also observed in the same kidney. The patient underwent laparoscopic left radical nephrectomy, which revealed a renal high-grade NET (NET G1) with old hemorrhage and cystic changes limited to the renal capsule, without infiltration of nearby tissues or structures. Immunohistochemistry showed various markers, including CK-pan (scattered +), Vimentin (+), PAX-8 (-), P504S (partially +), CD10 (-), CgA (focal +), Syn (+), TTF-1 (-), CD117 (focal +), Ki-67 (+1%), SSTR2 (+), P53 (weak +20%), and CK7 (-). Adrenal tissue was observed peripherally without tumor infiltration, while lymph node metastasis was observed in the renal hilum (1/2). The patient is recovering well postoperatively and has been followed up closely with no evidence of metastasis on a PET-CT scan performed three months after surgery.

We conducted a literature search in the PubMed database to retrieve relevant articles on cases of renal well-differentiated NETs, previously referred to as renal carcinoids. Our search strategy included the following search terms: “carcinoid [title] and kidney [title],” “carcinoid [title] and renal [title],” “Renal[Title] and Neuroendocrine[Title],” and “(kidney[Title]) AND (Neuroendocrine[Title])”. We specifically focused on articles written in the English language. Our analysis aimed to investigate the clinical manifestations, age distribution, gender distribution, tumor size, location, gross pathology, histology, and immunohistochemistry of renal well-differentiated NETs.

The trial was conducted in accordance with the principles of the Declaration of Helsinki. Written informed consent for the publication of this report was obtained from the patient.

## 3 Results

In our case, the patient recovered well after surgery and was discharged three days post-operation. During follow-up, the patient did not present with symptoms of carcinoid syndrome, such as flushing, abdominal pain, wheezing, or diarrhea. A PET-CT scan performed three months post-operation did not reveal any recurrence or distant metastasis. A review of relevant English literature revealed that Romero et al. (1) summarized 56 cases of renal carcinoids up to 2006, Korkmaz et al. (2) reviewed 26 cases of primary renal carcinoids from 2006 to 2012, and Jiang et al. (3) reviewed 29 cases from 2013 to 2020. We identified a total of 32 cases reported in English literature after these reviews, including the one we report here. We found that renal well-differentiated NETs lack typical clinical manifestations and are mainly characterized by back pain, abdominal distension, abdominal mass, and hematuria. There is no significant difference in median age between males and females. Although renal well-differentiated NETs have low malignancy, they are prone to distant metastasis, local invasion, and lymph node metastasis in the early stages of the disease. Renal well-differentiated NETs are often associated with horseshoe kidneys and renal teratomas. In terms of diagnosis, renal well-differentiated NETs lack specificity in imaging studies, but renal well-differentiated NETs specifically express neuroendocrine markers such as Syn, CD56, and CgA. These markers have high specificity and sensitivity for diagnosing renal well-differentiated NETs. Currently, surgery remains the primary treatment for renal well-differentiated NETs. However, some cases may experience systemic multiple metastases several years after nephrectomy. Therefore, patients require lifelong follow-up every three months, even if the tumor cells are well-differentiated, low-grade, or in the early stages of the disease.

Clinical features of renal well-differentiated NETs are shown in **Table 1**. Pathological features and pathological stages are shown in **Table 2**. Immunohistochemical results of these cases are shown in **Table 3**.

**Table 1:** Clinical features of the patients.

Reference	Gender	Age	Symptoms	HSK/MT	Treatment	Metastatic Site	Follow up (Months)
**Amin et al (** 14)	F	58	NS	No	PN	NED	60
	F	44	NS	No	PN	NED	36
	F	45	NS	No	RN+ Targeted Therapy+ somatostatin analogues	Bones, after 60 months	60
	F	51	NS	No	RN+ Targeted Therapy + somatostatin analogues	After 60 months, breast, brain, bones	60
	F	60	NS	No	RN+ Targeted Therapy	Liver, after 60 months	60
**Chen et al (** 37)	F	59	Abdominal pain	No	PN	Liver, after 60 months	NS
	M	66	Hematuria	No	RN	Liver	8
	F	58	hematuria /flank pain	No	RN	NED	NS
**Deacon et al.** (15)	F	36	Abdominal pain/ back pain	No	RN+ somatostatin analogues	Liver. After 2 months, bilobar liver and pelvic skeletal	6
**Gupta et al (** 38)	F	late 40s	Hematuria	HSK	PN	NED	NS
**Kelly et al (** 39)	F	63	Incidental	No	RN	NED	30
	F	53	Abdominal pain	No	PN	NED	6
**Ungerer et al (** 40)	M	41	Incidental	MT	PN	NED	6
**Paisey et al (** 16)	F	51	Flank/back pain	No	RN+adjuvant chemotherapy	Liver after surgery	NS
	F	36	Abdominal/back pain	No	RN+ somatostatin analogues	Bilobar liver and pelvic skeletal after surgery	NS
	M	68	Back pain	No	RN	NED	NS
**Prasad et al (** 41)	F	48	Incidental	No	RN	NED	12
**Yang et al (** 42)	F	45	Flank pain	No	PN	NED	12
**Yin et al (** 43)	F	33	Incidental	No	RN	NED	NS
**Kasajima et al (** 13)	M	50	NS	NS	NS	NED	24
	F	39	NS	NS	NS	NS	NS
	M	42	NS	NS	NS	Lymph node	60
	F	27	NS	NS	NS	Lymph node	58
	F	50	NS	NS	NS	Liver, bone, soft tissue	17
	F	28	NS	NS	NS	Lymph node	NS
	F	43	NS	NS	NS	Liver	63
	F	52	NS	NS	NS	Lymph node	96
	M	63	NS	NS	NS	NED	63
	M	31	NS	NS	NS	NED	55
	F	42	NS	NS	NS	Lymph node	12
	F	32	Cushing syndrome	NS	NS	NED	48
	F	54	Cushing syndrome	NS	NS	NED	57
**Our case**	**F**	**55**	**Incidental**	**No**	**RN**	**NED**	**3**

M, Male; F, female; L, left; R, right; LN, lymph nodes; FNA, fine needle aspiration; RN, radical nephrectomy; PN, partial nephrectomy; HSK; horseshoe kidney; MT, mature teratoma; NS, not stated; NED, no evidence of disease.

**Table 2:** Pathologic features of the patients.

Reference	Side	Size(cm)	Gross Pathology	Pathologic Stage
**Amin et al (** 14)	L	3.4	NS	G1
	R	4.5	NS	G1
	R	4.5	NS	G2
	L	14.0	NS	G2
	R	9.0	NS	G2
**Chen et al (** 37)	R	6.0	Cystic-solid	NS
	R	6.0	NS	G2
	R	8.0	Cystic-solid	NS
**Deacon et al.** (15)	R	14.0	Cystic-solid	G2
**Gupta et al (** 38)	NS	14.9	NS	NS
**Kelly et al (** 39)	L	3.5	NS	NS
	R	6.0	Solid	NS
**Ungerer et al (** 40)	L	1.5	Cystic-solid	G1
**Paisey et al (** 16)	NS	9.5	NS	G2
	R	15.5	Solid	G2
	R	7.0	NS	G2
**Prasad et al (** 41)	R	5.7	Cystic-solid	G1
**Yang et al (** 42)	R	7.0	Solid	G2
**Yin et al (** 43)	L	4.6	NS	G1
**Kasajima et al (** 13)	L	8.5	NS	G1
	L	12.5	NS	G1
	L	7.0	NS	G2
	R	7.5	NS	G2
	R	5.0	NS	G2
	L	11.0	NS	G2
	L	8.8	NS	G2
	R	5.9	NS	G2
	R	8.0	NS	G2
	R	6.2	NS	G3
	R	8.0	NS	G3
	R	4.5	NS	G1
	R	3.6	NS	G3
**Our case**	**L**	**10**	**Cystic-solid**	**G1**

L, left; R, right; NS, not stated.

The vertically bolded items in the "Reference" column refer to the corresponding references cited in the article.The horizontally bolded items in the last row represent the case reported in our study.

**Table 3:** Immunohistochemical features of the patients.

Reference	Syn	Cg A	NSE	CD56	CD10	CK7	CK20	TTF-1	Vim	Ki-67
**Amin et al (** 14)	+	NS	NS	NS	NS	–	–	NS	NS	2%
	+	NS	NS	NS	NS	NS	NS	NS	+	2%
	+	NS	NS	NS	NS	–	–	–	NS	13%
	+	NS	+	NS	NS	NS	NS	NS	NS	NS
	+	NS	NS	NS	NS	–	–	–	NS	4%
**Chen et al (** 37)	+	–	NS	NS	NS	NS	NS	NS	NS	NS
	+	+	NS	–	NS	NS	NS	NS	NS	10%
	+	–	NS	NS	NS	NS	NS	NS	NS	NS
**Deacon et al.** (15)	+	+	NS	+	–	–	NS	NS	NS	5%
**Gupta et al (** 38)	NS	NS	NS	NS	NS	NS	NS	NS	NS	NS
**Kelly et al (** 39)	+	NS	NS	+	NS	NS	NS	NS	NS	NS
	+	NS	NS	+	NS	NS	NS	NS	NS	NS
**Ungerer et al (** 40)	+	+	NS	NS	NS	–	NS	NS	NS	1%
**Paisey et al (** 16)	+	+	NS	+	NS	–	–	NS	+	10%
	NS	+	NS	+	–	–	–	–	–	5%
	+	+	NS	NS	–	+	NS	NS	+	10%
**Prasad et al (** 41)	+	–	NS	+	NS	NS	NS	NS	NS	2-3%
**Yang et al (** 42)	+	+	NS	+	NS	NS	NS	NS	NS	<10%
**Yin et al (** 43)	+	NS	NS	+	NS	–	NS	NS	NS	3%
**Kasajima et al (** 13)	NS	NS	NS	NS	NS	NS	NS	NS	NS	2%
	NS	NS	NS	NS	NS	NS	NS	NS	NS	2%
	NS	NS	NS	NS	NS	NS	NS	NS	NS	3%
	NS	NS	NS	NS	NS	NS	NS	NS	NS	4%
	NS	NS	NS	NS	NS	NS	NS	NS	NS	5%
	NS	NS	NS	NS	NS	NS	NS	NS	NS	5%
	NS	NS	NS	NS	NS	NS	NS	NS	NS	7%
	NS	NS	NS	NS	NS	NS	NS	NS	NS	7%
	NS	NS	NS	NS	NS	NS	NS	NS	NS	8%
	NS	NS	NS	NS	NS	NS	NS	NS	NS	21%
	NS	NS	NS	NS	NS	NS	NS	NS	NS	23%
	NS	NS	NS	NS	NS	NS	NS	NS	NS	2%
	NS	NS	NS	NS	NS	NS	NS	NS	NS	33%
**Our case**	**+**	**+**	**NS**	**NS**	**-**	**-**	**NS**	**-**	**+**	**1%**

Cg A, chromogranin A; Syn, synaptophysin; NSE, neuron specific enolase; TTF-1, Thyroid transcription factor-1; Vim, vimentin; NS, not stated; +, positive; -, negative.

The vertically bolded items in the "Reference" column refer to the corresponding references cited in the article.The horizontally bolded items in the last row represent the case reported in our study.

## 4 Discussion

### 4.1 Pathogenesis

Renal well-differentiated NETs, previously identified as renal carcinoids, represent a rare subset of neuroendocrine neoplasia (NEN). To date, over 160 cases of primary renal well-differentiated NETs have been documented in the literature. These neoplasms are marked by the presence of biomarkers linked to the regulated secretion pathways of normal neuroendocrine cells/neurons, especially those associated with large dense core vesicles (LDCV) and small synaptic-like vesicles (SSV) (4). Recently, insulinoma-associated 1 (IA1 or INSM1), a zinc finger transcription factor, has also been found to be expressed in NENs (5). NETs are further distinguished by their ability to produce various biologically active substances such as kinin, dopamine, histamine, prostaglandin, and 5-hydroxytryptamine. While NETs often result in carcinoid syndrome within the gastrointestinal and respiratory systems, only four cases of renal NETs have been reported (6).

The pathogenesis of renal NETs remains enigmatic, as neuroendocrine cells are not typically found in normal renal parenchyma. Azzopardi et al. noted that renal primordia can differentiate into argyrophilic cells within the tumor microenvironment (7). Renal NETs are frequently associated with developmental anomalies such as horseshoe kidneys (HSK) and mature cystic teratomas (MT). It is posited that early teratogenic events or genetic abnormalities leading to HSK may contribute to the development of malignant renal tumors, including teratomas (8). Patients with HSK have a relative risk of 62% to 82% for developing well-differentiated NETs (9, 10). Notably, all primary well-differentiated NETs originate in the renal isthmus of HSK, suggesting that this region may be prone to atypical cytological development, ultimately leading to the formation of NETs (1).

Data on the molecular and genetic background of primary renal NETs is limited. The most common genetic alteration observed in primary renal NETs is the heterozygous loss of chromosome 3p21. The mutation profile in renal NETs shows considerable variability, and no specific genes or affected pathways have been exclusively identified in renal NETs. The most frequently observed genetic mutations in renal NETs include alterations in CDH1 and TET2, as well as heterozygous loss of 3p and mutations in AKT3, ROS1, PIK3R2, BCR, and MYC. Several of these frequently mutated genes implicate the PI3K-AKT signaling pathway (11).

### 4.2 Clinical manifestations and epidemiology

Primary renal NETs often present with nonspecific clinical symptoms and are typically detected incidentally during routine physical examinations. When symptomatic, patients may report abdominal or back pain, hematuria, or signs of metastatic disease (**Table 1**). Rarely, patients may exhibit carcinoid syndrome with symptoms such as 5-hydroxytryptamine-related flushing, systemic edema and diarrhea, and occasionally elevated levels of 5-hydroxyindoleacetic acid in the urine. However, the incidence of carcinoid syndrome is much lower in renal NETs than in gastrointestinal and respiratory NETs, with only four reported cases in the literature (3). This disparity in occurrence may be attributed to the hindgut origin of renal NETs, as well as the degradation of their secreted biologically active hormones in the liver before they can reach the systemic arterial circulation (12). The research conducted by Kasajima et al. recently demonstrates that most renal NETs exhibit a distinct reticulated trabecular morphology and consistently coexpress ISL1 and SATB2. These tumors are typically non-functioning, although they do express a variety of entero-pancreatic hormones. Additionally, there are NETs that express ACTH without the presence of ISL1 or SATB2, exhibiting a typical solid eosinophilic cell morphology. Patients with such tumors often present with ectopic Cushing syndrome (13).

Although renal well-differentiated NETs are generally low-grade malignant tumors, they often exhibit local invasion, lymph node metastasis, and distant metastasis in the early stages. In this study, nine cases (27%, 9/33) exhibited local invasion involving the renal sinus, perirenal fat, and nerve involvement, while six cases (33%, 11/33) had lymph node metastasis, and four cases (12%, 4/33) had distant metastasis, all of which were liver metastases. After surgery, tumors also metastasized to distant sites such as breast, brain, bones, bilobar liver and pelvic skeletal, highlighting the importance of regular long-term follow-up for patient prognosis (14–16).

Previous studies have shown that unlike clear cell renal cell carcinoma, high-grade renal neuroendocrine carcinoma appears to occur earlier and is diagnosed around the age of 49 years with no gender differences (17, 18). In this study, patients were diagnosed between the ages of 27 and 68 years. Regarding gender differences, the sample size in this study was small, with 26 female cases and 7 male cases.

### 4.3 Classification

The 2022 WHO Classification of Neuroendocrine Neoplasms adopts a 3-tiered grading system for NETs in various organs, based on proliferation rates determined by mitotic count and Ki67 labeling index. This system categorizes NETs into grade 1 (G1), grade 2 (G2), and grade 3 (G3), corresponding to low, intermediate, and high grades. However, no specific staging system exists for renal NETs, and the TNM system for renal cell carcinoma is unsuitable (6). Some scholars advocate for the WHO (1999) classification for pulmonary NETs as a more accurate predictor of the biological behavior of renal NETs (19). Research by Amin et al. emphasizes the importance of the Ki-67 index and mitotic count for classification, recommending that all renal NENs be graded according to the criteria established for gastroenteropancreatic NENs (14).

### 4.4 Imaging

**Figure 1:**
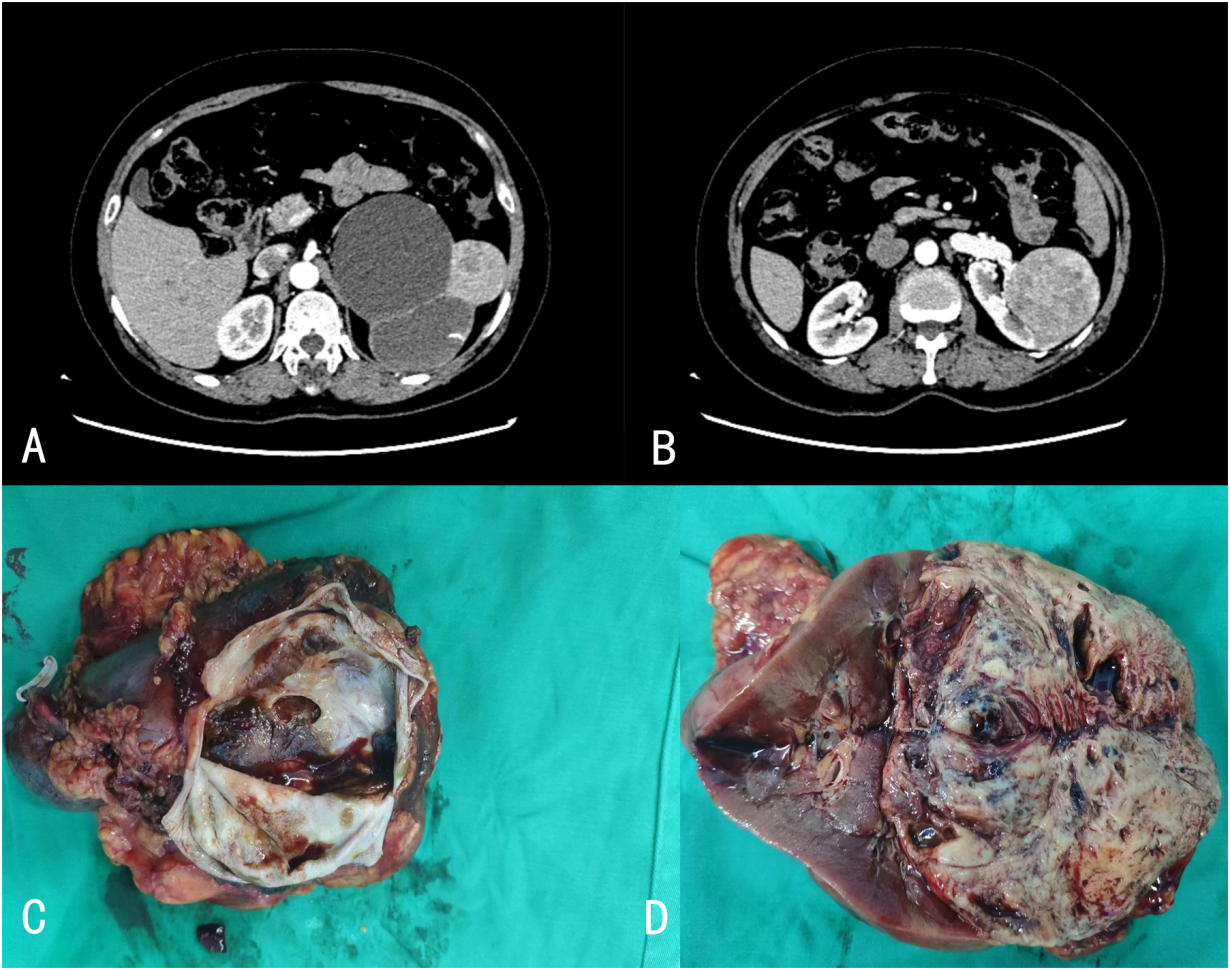
**(A, B)** Enhanced CT revealed a heterogeneous soft tissue density mass in the left kidney, with significant heterogeneous enhancement during the arterial phase, decreased density during the venous and pyelographic phases, and non-enhanced areas within, measuring approximately 6.9 × 7.1 cm in size. A low-density round lesion with Bosniak classification of IIF was also observed in the same kidney. **(C, D)** The postoperative pathology revealed the presence of a mass in the upper pole of the kidney, measuring 10 cm × 9.5 cm × 6.5 cm in size, with a cystic-solid appearance on cross-section. The solid area accounted for 60% of the mass, with a pale yellow, solid, and soft texture. Two cystic cavities, measuring 6 cm and 8 cm in diameter, were observed within the cystic area.

Preoperative imaging of renal NETs typically does not reveal specific features that distinguish them from other renal tumors on computed tomography (CT) or magnetic resonance imaging (MRI). Generally, well-differentiated renal NETs present as solid masses with well-defined margins, showing either no enhancement or minimal enhancement. However, cystic components or calcifications may occasionally be observed (20). Renal angiography often reveals these lesions as hypovascular or avascular (21). In the case discussed, CT imaging revealed significant heterogeneous enhancement during the arterial phase, a reduction in density during the venous and pyelographic phases, and non-enhanced regions within the tumor. Additionally, a low-density round lesion with a Bosniak classification of IIF was identified in the same kidney (**Figures 1A, B**). Octreotide scintigraphy has proven to be a sensitive modality for diagnosing, staging, and monitoring recurrence or metastasis of NETs (22). Although nuclear medicine techniques have been extensively utilized for gastrointestinal NETs, the normal renal uptake of tracer substances can obscure lesions when using octreotide scintigraphy or positron emission tomography (PET) with fludeoxyglucose (FDG) in the evaluation of primary renal NETs. The low FDG affinity in low-grade NETs further diminishes the effectiveness of PET imaging in these cases. The reduced sensitivity of FDG-PET in detecting metastases of primary renal NETs is likely due to their low metabolic activity and indolent growth (2). Yakemchuk et al. have reported that 18F-Dopa PET/CT offers superior diagnostic value over MRI alone in the assessment of renal NETs (23).

### 4.5 Pathology and immunohistochemistry

Renal NETs typically manifest as solitary, well-defined masses with a lobulated, protruding appearance. Gross examination reveals a solid or cystic-solid consistency, commonly presenting in shades of yellow-brown, beige, or reddish-brown (24). In the case under study, the tumor exhibited a cystic-solid consistency, with approximately 60% of the cross-sectional area being solid. The solid portion was pale yellow, solid, and soft in texture, while the cystic region contained two cavities measuring 6 cm and 8 cm in diameter. The larger cavity had a gray-white to light brown inner wall with a slightly rough texture, whereas the smaller cavity showed diffuse papillary projections on the inner wall, with diameters ranging from 0.3 to 3 cm (**Figures 1C, D**). Statistical analysis of well-differentiated NETs has shown an average tumor diameter of 7.73 cm, with a range spanning from 1.5 to 15.5 cm (**Table 2**).

**Figure 2:**
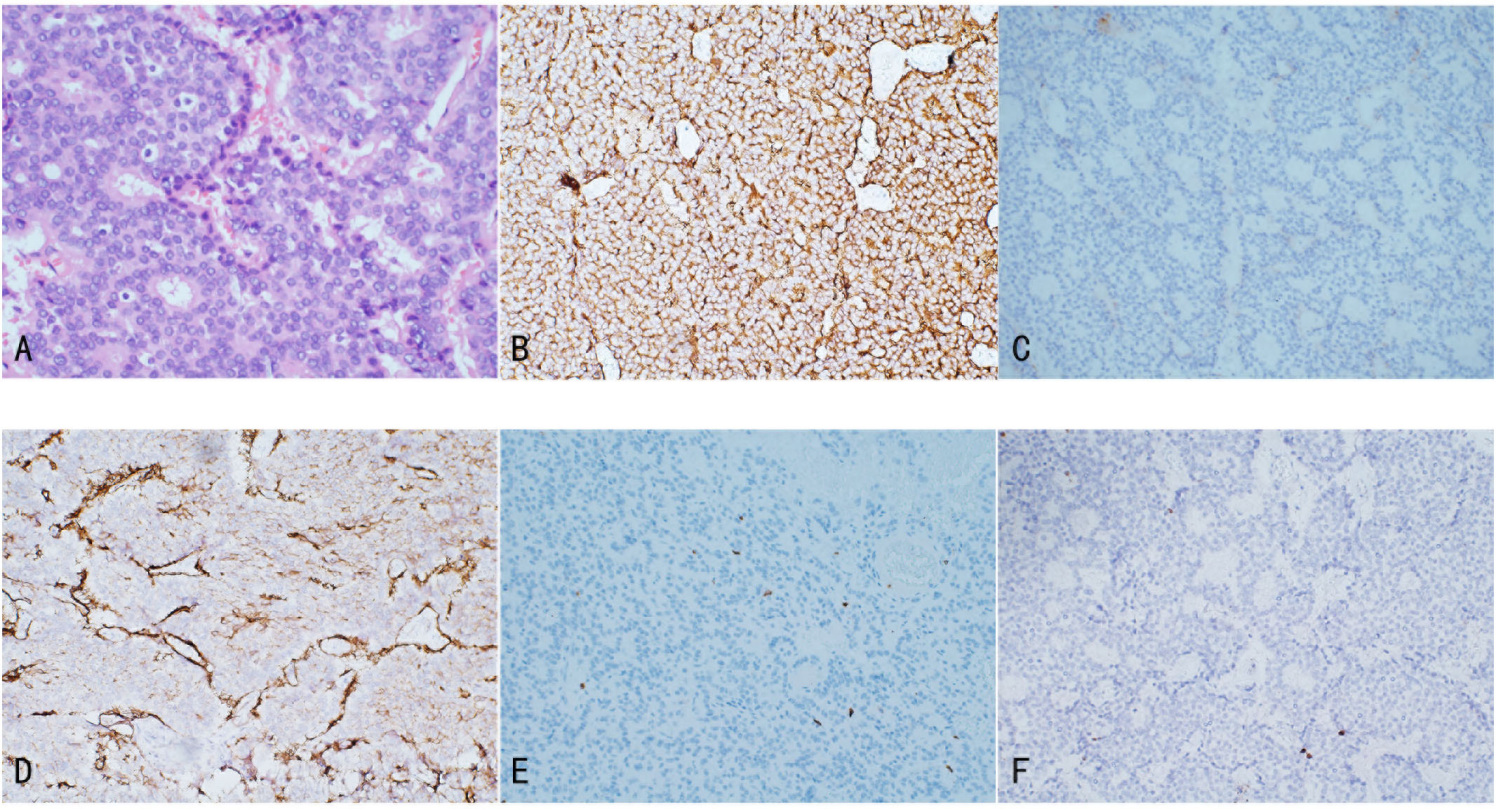
Illustrates the pathological features of Renal NETs: **(A)** H&E staining reveals nests and trabecular arrangements of neuroendocrine cells, forming a rosette-like structure. **(B)** Tumor cells exhibit strong positivity for Syn, with abundant membrane-bound electron-dense neurosecretory granules. **(C)** Focal positivity for CgA is observed. **(D)** SSTR2 shows positive staining. **(E)** CD10 staining is negative. **(F)** Ki-67 expression is 1%.

Histopathologically, renal NETs share common characteristics with NETs occurring in other organs, including distinct separation from the surrounding renal parenchyma. Tumor cells demonstrate a variety of morphologies, such as round, cuboidal, columnar, or polygonal shapes, with granular, eosinophilic cytoplasm. The nuclei are typically elliptical, and the cells are organized into small cords or ribbon-like structures, interspersed with solid nests and cell clusters composed of monotonous cells. The chromatin pattern is characterized by the classic “salt and pepper” appearance typical of NETs (**Figure 2A**) (25). Additionally, necrotic foci, cellular atypia (including nuclear pleomorphism and increased chromatin), hemorrhage, or calcification may be present. Necrosis and hemorrhage suggest a more aggressive tumor behavior, while calcification may indicate a long-standing lesion or a teratomatous component. In rare instances, metaplastic bone formation can be observed within the tumor, as reported by Hansel et al. (25). Due to the rarity of renal NETs and the nonspecific nature of their clinical and imaging features, histopathological diagnosis can be challenging and is sometimes inconclusive. Scanning electron microscopy (SEM) of biopsy specimens may assist in diagnosis by revealing numerous electron-dense core neurosecretory granules in the cytoplasm (26).

Immunohistochemistry is a critical tool for the diagnosis of renal NETs, primarily based on the detection of specific markers common to neuroendocrine tissue. The most valuable immunohistochemical markers include Chromogranin A (CgA), synaptophysin (Syn), neuron-specific enolase (NSE), and CD56. Among these, synaptophysin is considered a more sensitive marker for renal NETs than Chromogranin A or other markers such as CD56 and NSE. For instance, Korkmaz et al. reported an 88% sensitivity for synaptophysin compared to 61% sensitivity for Chromogranin A (2, 11). In our study, synaptophysin and Chromogranin A showed sensitivities of 100% (18/18) and 72.7% (8/11), respectively. Immunohistochemistry is also valuable in excluding other primary or metastatic tumors of the kidney. Markers such as Cytokeratin 7 and 20 are typically used to identify most primary and metastatic adenocarcinomas, while TTF-1 is a tissue-specific transcription factor mainly expressed in thyroid cancer, lung cancer, and high-grade neuroendocrine carcinomas, with minimal expression in extrapulmonary tumors. Negative staining for TTF-1, CK-7, and CK-20 typically excludes primary lung and intestinal origins. Moreover, negative WT1 staining helps exclude Wilms tumor, and negative CD10 staining excludes renal cell carcinoma and urothelial carcinoma. The Ki-67 antigen, a marker of cell proliferation, is used to grade NENs. According to the WHO classification, Ki-67 typically being highly expressed (>55%) in poorly differentiated NEC. In the case under review, tumor cells exhibited strong positivity for synaptophysin, with abundant membrane-bound electron-dense neurosecretory granules. Focal positivity for Chromogranin A was also observed, and the Ki-67 index was only 1%, indicating low proliferative activity (**Figure 2**) (6, 27).

### 4.6 Treatment and prognosis

Radical nephrectomy remains the cornerstone of treatment for primary renal well-differentiated NETs. Depending on the tumor’s location and size, partial nephrectomy may also be an option. However, current evidence does not suggest that radical nephrectomy offers a significant advantage over partial nephrectomy. Lymph node dissection during surgery is recommended for optimal staging. As of now, no systemic therapies have been established for either adjuvant or metastatic settings in renal NETs. While the RADIANT trials have demonstrated the efficacy of everolimus in patients with lung and gastroenteropancreatic NENs, there is limited data on the use of mammalian target of rapamycin (mTOR) inhibitors in renal NETs (28, 29). Research suggests that clear cell renal cell carcinoma (CCRCC) originates from the renal erythropoietin-producing cell (EPC), which shows markers consistent with a neuroendocrine origin (30). In one study, NSE was expressed in 97% of CCRCC, with 9% expressing synaptophysin, suggesting that CCRCC originates from renal EPC, indicating a potential origin from renal EPC. Further studies are needed to explore whether EPCs are the origin cells for renal NENs (31). Additionally, molecular abnormalities similar to those found in renal cell carcinomas have been reported in some renal NETs (32). Some renal NETs also exhibit features and morphology resembling CCRCC, including erythrocytosis due to erythropoietin overproduction, early metastasis, and in some cases, apparent dormancy with late recurrence (33). Recent advances in the treatment of kidney cancer, including checkpoint inhibitors and PDL1 inhibitor combination therapies, have shown considerable promise. However, these therapeutic approaches still need to be evaluated in renal NENs. In a study by Amin et al., patients with poorly differentiated tumors were treated with systemic chemotherapy, including etoposide and platinum-based agents (cisplatin/carboplatin). In contrast, patients with well-differentiated histology received somatostatin analogues, everolimus, and supportive therapies, such as bisphosphonates or denosumab, for bone metastatic disease (14). Octreotide, a somatostatin analogue, has been widely used in treating various NETs. Although non-functioning NETs do not secrete active hormones, both functional and non-functional NETs typically express somatostatin receptors, as determined through somatostatin receptor scintigraphy using a radiolabeled somatostatin analogue. Octreotide is considered a first-line antitumor systemic therapy for octreotide-positive patients, in addition to reducing symptoms of hormone excess (2). A study by Rinke et al. showed that octreotide had a significant anti-proliferative effect on metastatic neuroendocrine midgut tumors (34). Long-term use of octreotide in metastatic renal NETs has also been associated with favorable clinical outcomes (35).

Due to the rarity of renal NETs and the limited availability of long-term follow-up data, the prognosis and clinical behavior of these tumors remain unclear. However, three major prognostic factors have been identified. Patients over 40 years old tend to have more rapidly progressing disease and more severe initial symptoms. Tumors with a maximum diameter of less than 4 cm or those confined to the renal parenchyma generally have fewer metastases and a better prognosis. Other significant prognostic factors include a high mitotic rate (greater than 2/10 high-power fields), atypical cytology, lymphovascular invasion, and the presence of necrosis (2). Romero et al. found that 47% of patients had lymph node metastases, with an average disease-free survival of 43 months. However, patients with metastases to the bones, liver, and contralateral kidney had a poor prognosis, with overall survival times of only a few months (1). One patient reported by Rodriguez-Covarrubias et al. survived more than three years despite distant organ metastases at diagnosis, whereas another patient reported by Korkmaz et al. died 11 months after diagnosis (2, 36). In our series, the longest follow-up time was 60 months, the shortest was 3 months, and there were no deaths.

Close postoperative follow-up is crucial. Postoperative imaging should include somatostatin receptor scintigraphy to detect metastatic disease, given the non-specificity of CT and MRI. Long-term follow-up is recommended since metastatic disease may occur even after five years from diagnosis. Follow-up should include physical examination, biochemical laboratory tests, and Chromogranin A (CgA) levels, along with imaging studies every 3-6 months.

## 5 Conclusion

Primary renal well-differentiated NETs are exceedingly rare and typically present as low-grade malignancies with a favorable prognosis. Key diagnostic markers include CgA, Syn, NSE, and CD56, which are critical for accurate diagnosis. Surgical resection remains the primary treatment modality, with octreotide showing potential as an effective adjuvant therapy in cases of metastatic disease. However, due to the rarity of these tumors, ongoing research and extended follow-up are essential to better understand their biological behavior and to develop more effective treatment strategies.
